# Leukemia Inhibitory Factor-Receptor is Dispensable for Prenatal Testis Development but is Required in Sertoli cells for Normal Spermatogenesis in Mice

**DOI:** 10.1038/s41598-018-30011-w

**Published:** 2018-08-01

**Authors:** Michael Curley, Laura Milne, Sarah Smith, Nina Atanassova, Diane Rebourcet, Annalucia Darbey, Patrick W. F. Hadoke, Sara Wells, Lee B. Smith

**Affiliations:** 1 MRC Centre for Reproductive Health, University of Edinburgh, The Queen’s Medical Research Institute, 47 Little France Crescent, Edinburgh, EH16 4TJ United Kingdom; 2 Institute of Experimental Morphology, Pathology and Anthropology with Museum, Bulgarian Academy of Sciences, 1113 Sofia, Bulgaria; 3 The British Heart Foundation Centre for Cardiovascular Science, University of Edinburgh, The Queen’s Medical Research Institute, Edinburgh, EH16 4TJ United Kingdom; 4 Mary Lyons Centre, MRC Harwell, Harwell Campus, Oxfordshire, OX11 ORD United Kingdom; 5 School of Environmental and Life Sciences, University of Newcastle, Callaghan, NSW 2308 Australia

## Abstract

Leukemia inhibitory factor (LIF), a pleiotropic cytokine belonging to the interleukin-6 family, is most often noted for its role in maintaining the balance between stem cell proliferation and differentiation. In rodents, LIF is expressed in both the fetal and adult testis; with the peritubular myoid (PTM) cells thought to be the main site of production. Given their anatomical location, LIF produced by PTM cells may act both on intratubular and interstitial cells to influence spermatogenesis and steroidogenesis respectively. Indeed, the leukemia inhibitory factor receptor (LIFR) is expressed in germ cells, Sertoli cells, Leydig cells, PTM cells and testicular macrophages, suggesting that LIF signalling *via* LIFR may be a key paracrine regulator of testicular function. However, a precise role(s) for testicular LIFR-signalling *in vivo* has not been established. To this end, we generated and characterised the testicular phenotype of mice lacking LIFR either in germ cells, Sertoli cells or both, to identify a role for LIFR-signalling in testicular development/function. Our analyses reveal that LIFR is dispensable in germ cells for normal spermatogenesis. However, Sertoli cell LIFR ablation results in a degenerative phenotype, characterised by abnormal germ cell loss, sperm stasis, seminiferous tubule distention and subsequent atrophy of the seminiferous tubules.

## Introduction

The mammalian testis is a complex multicellular organ, separated into two distinct compartments which carry out its principle functions. In the adult testis, sperm production (spermatogenesis) occurs within the seminiferous tubules, and androgen biosynthesis (steroidogenesis) occurs in Leydig cells found in the interstitial space. Both these processes are subject to tight regulation at endocrine and paracrine levels. In addition to negative feedback control of testicular function by the hypothalamic-pituitary-gonadal (HPG) axis, the importance of cross-talk between different cell types within the testis, required for the support of spermatogenesis and steroidogenesis, is well established^1,2^. For example; Leydig cell-derived androgens, signalling *via* androgen receptors in Sertoli cells and peritubular myoid cells, are essential for the maintenance of spermatogenesis^3–6^ whilst Sertoli cells, peritubular myoid cells and testicular macrophages have been shown to support Leydig cell development and steroidogenesis^7–12^. However, the full extent of the paracrine network which supports testicular function remains to be established. Identification of paracrine factors and/or mechanisms which regulate testicular function will be of benefit to the development of novel treatments for infertility and hypogonadism as well as for male contraceptive strategies.

Locally produced growth factors and cytokines have been suggested to play a role in the regulation of normal testicular development and function^1,13^. One such example is leukemia inhibitory factor (LIF) which belongs to the multifunctional interleukin-6 (IL-6)-related family of cytokines^14^. LIF signalling is mediated by a heterodimeric receptor complex consisting of the leukemia inhibitory factor receptor (LIFR, also known as gp190), which binds LIF, and the signal transducing gp130 subunit common to the IL-6 family members^15–17^. Expression of both LIF and LIFR, as well as the gp130 signal transducer, has been detected in the rodent testis from fetal stages through to adulthood, suggesting LIF/LIFR signalling may play a role in normal testicular development and function^18–21^. Peritubular myoid cells have been identified as the principal source of LIF within the rat testis and, given the anatomical location of these cells, LIF has been hypothesised to be a paracrine regulator of both the tubular and interstitial compartments^19^. Interestingly, LIF-deficient males are reported to be fertile^22^ whereas complete knockout of the LIFR results in perinatal death due to pleiotropic defects including neurological and metabolic disturbances^23^. Whilst LIFR is expressed by somatic Sertoli cells, Leydig cells, peritubular myoid cells and macrophages, spermatogonia have been speculated to be the main target of LIFR signalling within the rat testis. This supposition is based on *in vitro* binding assays with biotinylated LIF and immunohistochemical detection of LIFR in testis sections^18^; however, the precise role(s) of testicular LIFR signalling remains to be established.

To definitively identify the role(s) of LIFR signalling in the testis *in vivo*, we generated multiple cell-specific LIFR-deficient mice and analysed their testicular phenotype to dissect out and characterise the role of LIFR signalling in the development and function of the testis. Firstly, to determine whether LIFR signalling is required for prenatal testis development, we validated a novel *Lifr* knockout allele (*Lifr*^*tm1b(EUCOMM)Hmgu*^) and assessed the impact of complete LIFR-loss on testicular structure in new-born mice at postnatal day 0. We next generated testis cell-specific *Lifr*-knockout mice to identify potential role(s) for LIFR signalling in the postnatal/adult testis. The data presented herein demonstrate that (i) LIFR is dispensable for prenatal testis development and (ii) in adulthood, LIFR is required in Sertoli cells, but not developing germ cells, for the maintenance of normal spermatogenesis.

## Results

### LIFR is Not Required for Prenatal Testicular Development

**Figure 1:**
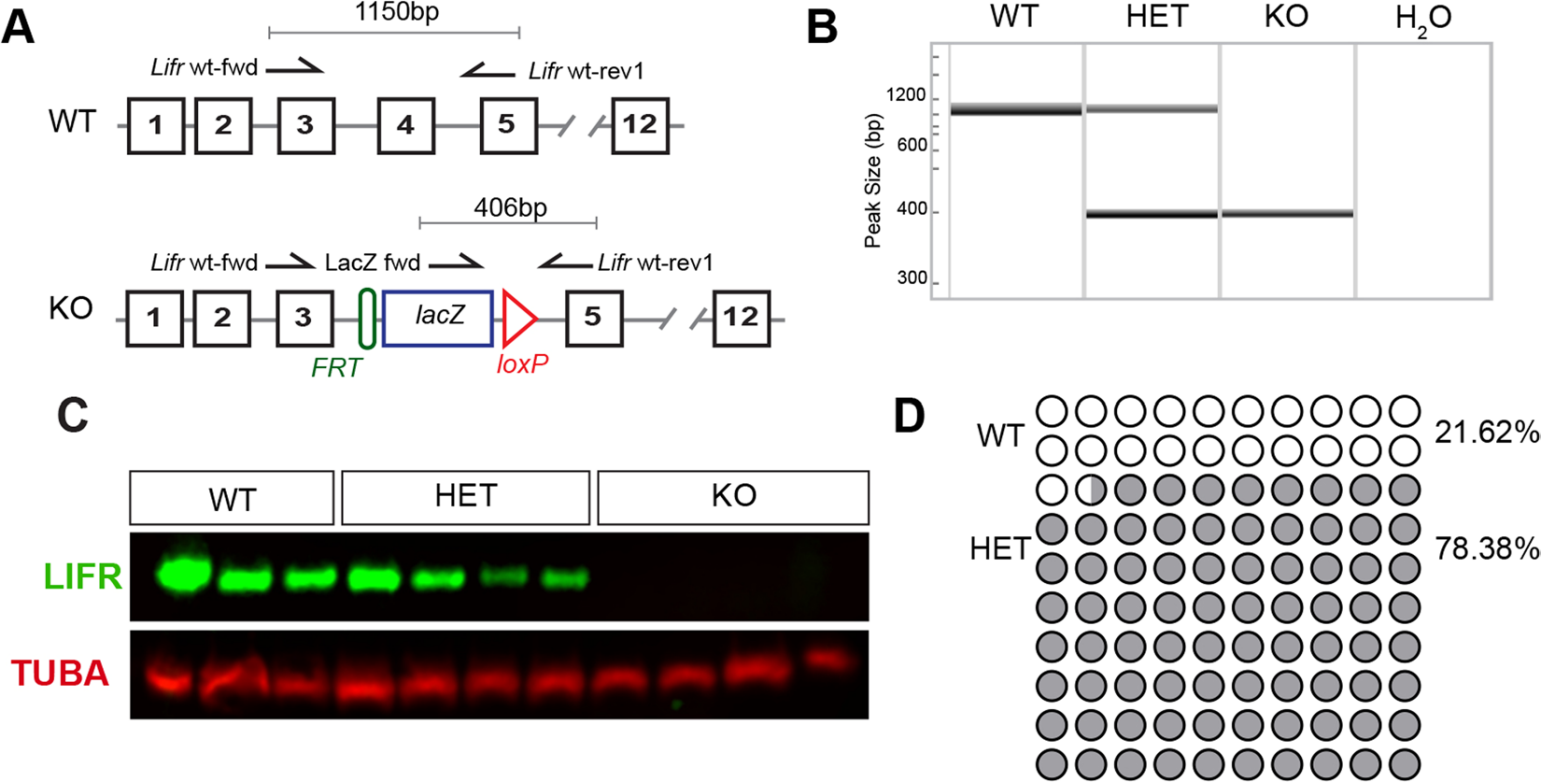
Validation of the *Lifr-*knockout allele. **(A)** Schematic of WT and KO alleles detailing the location of the genotyping primers and expected PCR product sizes. **(B)** Representative PCR analysis of genomic DNA isolated from tail-tip biopsies of neonatal mice identified wild-type (WT), heterozygous (HET) and homozygous (KO) animals. **(C)** Western blot analysis of neonatal brain tissue homogenates confirmed LIFR protein expression was abolished in KO animals. Tubulin-alpha (TUBA) was used as a loading control. Both LIFR and TUBA bands have been cropped from a single gel blot image which is included in the supplementary materials with the cropped areas highlighted. **(D)** Transgene inheritance in offspring derived from heterozygous matings based on genomic PCR of DNA isolated from ear-clip biopsies collected at weaning. Chi-squared analysis revealed a significant deviation from the expected Mendelian ratios (*X*^*2*^; *p* < 0.0001).

**Figure 2:**
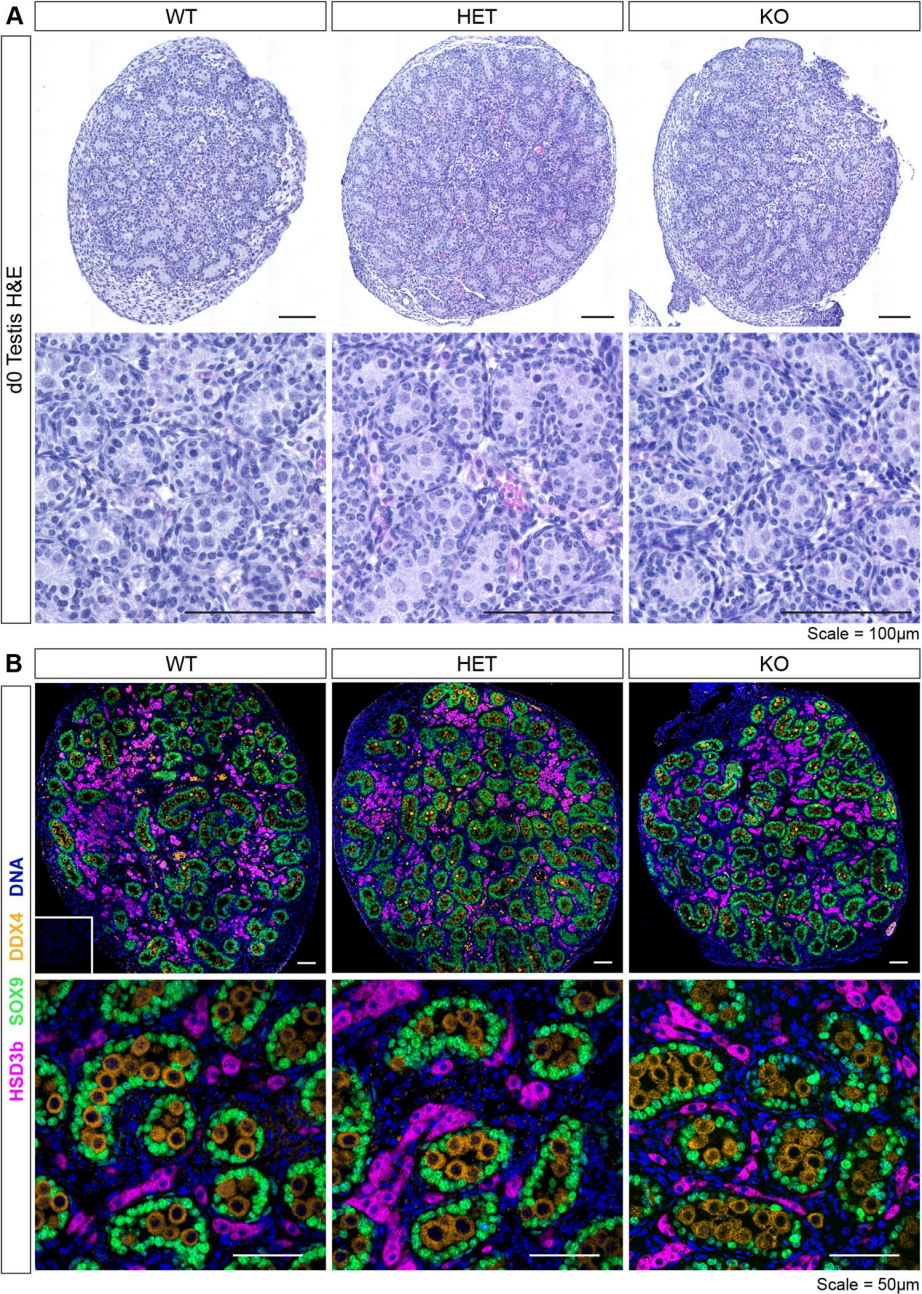
LIFR signalling is not required for prenatal testis development. **(A)** Representative H&E stained testis sections from WT, HET and KO mice at post-natal day 0. Testes appeared morphologically normal, with fully formed seminiferous cords containing abundant spermatogonia. n = 3–5, scale = 100 µm. **(B)** Immunostaining for HSD3b (magenta), SOX9 (green) and DDX4 (gold) confirmed the presence of fetal Leydig cells, Sertoli cells and spermatogonia respectively, No obvious difference was noted between WT, HET and KO testes. Inset = primary antibody negative control. n = 3–5, scale = 50 µm.

To determine whether there is a requirement for LIFR signalling in prenatal testis development, we first confirmed the absence of LIFR protein in new-born *Lifr*^*tm1b(EUCOMM)Hmgu*^ mice. Wild-type (WT), heterozygous (HET) and homozygous (KO) mice were generated as described in the materials and methods. Genotyping primers were designed to detect the synthetic targeting cassette (Fig. 1A) and used to amplify genomic DNA isolated from tail tip biopsies to identify WT, HET and KO animals (Fig. 1B). Western blot analysis of neonatal whole brain protein extracts revealed that LIFR protein expression was completely abolished in homozygous *Lifr*^*tm1b(EUCOMM)Hmgu*^ mice on postnatal day (d) 0 (Fig. 1C). At weaning, on d21, we noted a significant deviation from the expected Mendelian genotype ratios (Fig. 1D), reflecting absence of *Lifr* KO pups, consistent with previous reports of pre-weaning lethality in *Lifr-*KO mice^23^, and indicating that the *Lifr*^*tm1b(EUCOMM)Hmgu*^ allele is a true loss of function allele. We next assessed the impact of LIFR ablation on development of the prenatal testis. Histological analysis revealed that testicular architecture was normal in LIFR-deficient animals at d0 (Fig. 2A). No differences in the immuno-localisation of SOX9, DDX4 and HSD3b in the testes of WT, HET and KO animals was observed (Fig. 2B), suggesting that establishment of the Sertoli, germ and fetal Leydig cell populations, and the structural arrangement of these cells in the testis occurs normally in fetal life in the absence of LIFR signalling.

### Generation of Germ Cell and Sertoli Cell specific *Lifr*-KO Mice

**Figure 3:**
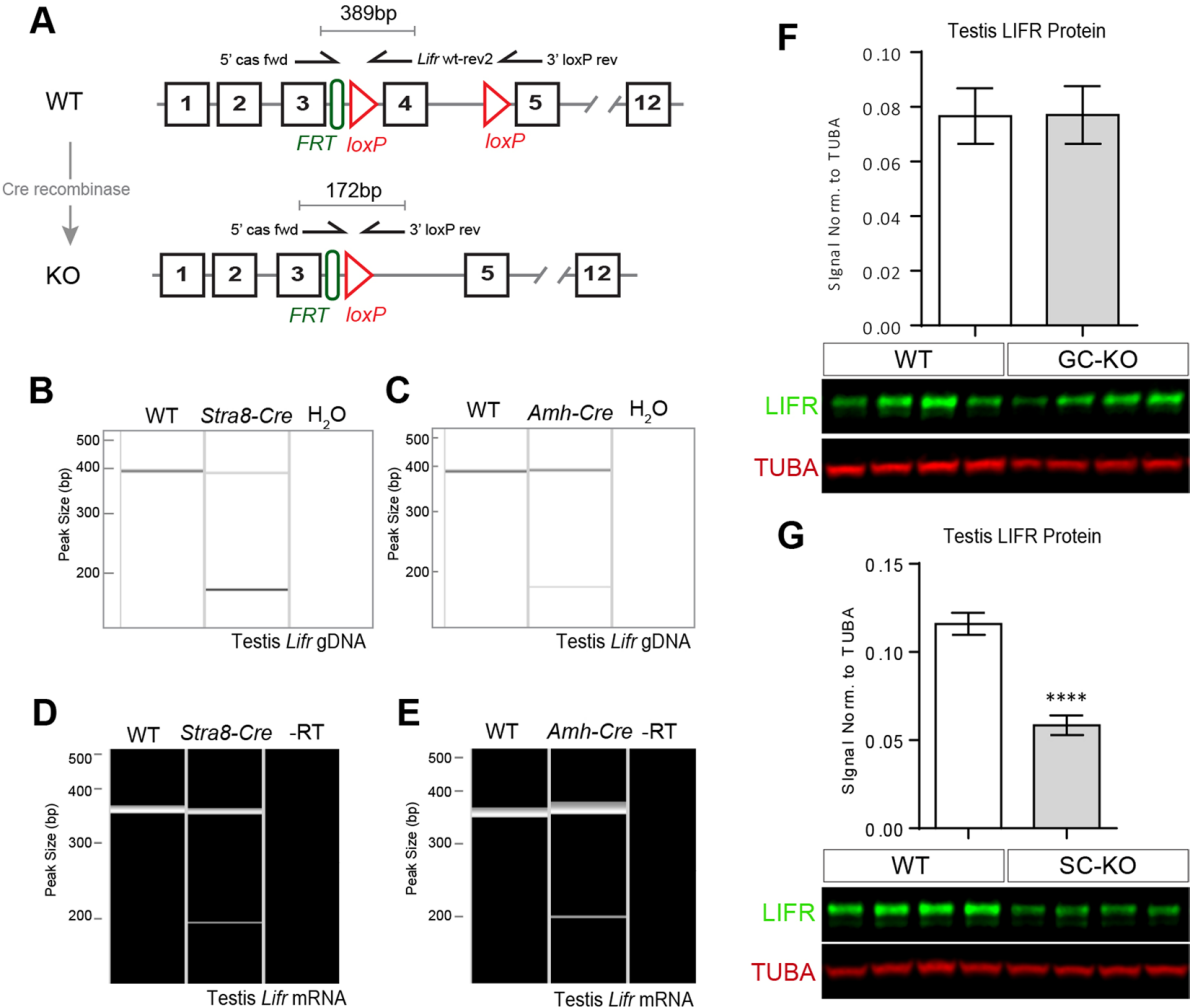
Generation of germ cell and Sertoli cell-specific *Lifr*-KO mice. **(A)** Schematic of the conditional *Lifr* allele detailing the location of PCR primers designed to detect Cre-mediated recombination and the expected sizes of the PCR products. Germ cell and Sertoli cell *Lifr*-knockout mice were generated as described in materials and methods. Representative PCR analysis of genomic DNA isolated from the testes of heterozygote *Stra8-Cre*^−/−^*;Lifr*^*wt/tm1c*^ and *Stra8-Cre*^+/−^*;Lifr*^*wt/tm1c*^ **(B)** and *Amh-Cre*^−/−^*;Lifr*^*wt/tm1c*^ and *Amh-Cre*^+/−^*;Lifr*^*wt/tm1c*^ **(C)** mice confirmed recombination of the conditional *Lifr* allele upon Cre exposure. Representative PCR analysis of testicular cDNA from *Stra8-Cre*^−/−^*;Lifr*^*wt/tm1c*^ and *Stra8-Cre*^+/−^*;Lifr*^*wt/tm1c*^ **(D)** and *Amh-Cre*^−/−^*;Lifr*^*wt/tm1c*^ and *Amh-Cre*^+/−^*;Lifr*^*wt/tm1c*^ **(E)** mice confirmed *Lifr* mRNA is expressed in germ cells and Sertoli cells, evidenced by the presence of a mutant transcript following Cre-mediated gDNA recombination. Representative Western blot analysis of whole testis protein extracts from *Stra8-Cre*^−/−^*;Lifr*^*tm1c/tm1c*^ (WT) and *Stra8-Cre*^+/−^*;Lifr*^*tm1c/tm1c*^ (GC-KO) **(F)** and *Amh-Cre*^−/−^*;Lifr*^*tm1c/tm1c*^ (WT) and *Amh-Cre*^+/−^*;Lifr*^*tm1c/tm1c*^ (SC-KO) **(G)** mice. A significant reduction in testicular LIFR protein is observed only when *Lifr* is disrupted in Sertoli cells (SC-KO; unpaired *t-*test; *p* = 0.001). For GC-KO and SC-KO blots, both LIFR and TUBA bands have been cropped from single gel blot images which are included in the supplementary materials with the cropped areas highlighted. Tubulin-alpha (TUBA) was used as a loading control. Values are expressed as mean ± S.E.M of n = 7 samples per genotype.

To circumvent the lethality observed in LIFR deficient animals, we utilised a conditional variant of the *Lifr* allele (*Lifr*^*tm1c(EUCOMM)Hmgu*^) to generate cell-specific LIFR knock-outs to identify potential role(s) for LIFR signalling in adult testis development/function. *Lifr* was selectively disrupted separately in germ cells (GC), Sertoli cells (SC) or both by breeding to the well-established *Stra8-*Cre^24^ and *Amh-*Cre^25^ lines. PCR analysis of genomic DNA isolated from the testes of heterozygote *Stra8*-Cre^+/−^; *Lifr*^*wt/tm1c*^ and *Amh*-Cre^+/−^; *Lifr*^*wt/tm1c*^ mice demonstrated recombination of the conditional *Lifr* allele upon exposure to either Cre-recombinase (Fig. 3A–C). Furthermore, PCR analysis of testicular cDNA revealed the presence of a mutant *Lifr* transcript following Cre-mediated recombination of *Lifr* gDNA, confirming that *Lifr* mRNA is expressed both in germ cells and in Sertoli cells (Fig. 3D,E respectively). According to the IMPC mutagenesis prediction, recombination of the *loxP* sequences flanking exon 4 of the *Lifr*^*tm1c(EUCOMM)Hmgu*^ allele is predicted to frameshift the *Lifr* transcript such that a premature stop codon is introduced into exon 5, resulting in no protein product. Interestingly, Western blot analysis of whole adult testis protein extracts from *Stra8-Cre*^+/−^*;Lifr*^*tm1c/tm1c*^ (GC-KO) and *Amh-Cre*^+/−^*;Lifr*^*tm1c/tm1c*^ (SC-KO) mice revealed a significant reduction of LIFR protein in SC-KO but not GC-KO testes compared to their respective controls (Fig. 3F,G). This suggests that, although *Lifr* mRNA is expressed in the germ cell population, the Sertoli cell population (which translates *Lifr* mRNA into LIFR protein) may be the major target of LIFR signalling within the seminiferous tubule.

### A Progressive, Degenerative Testicular Phenotype is observed in SC-KO Mice

**Figure 4:**
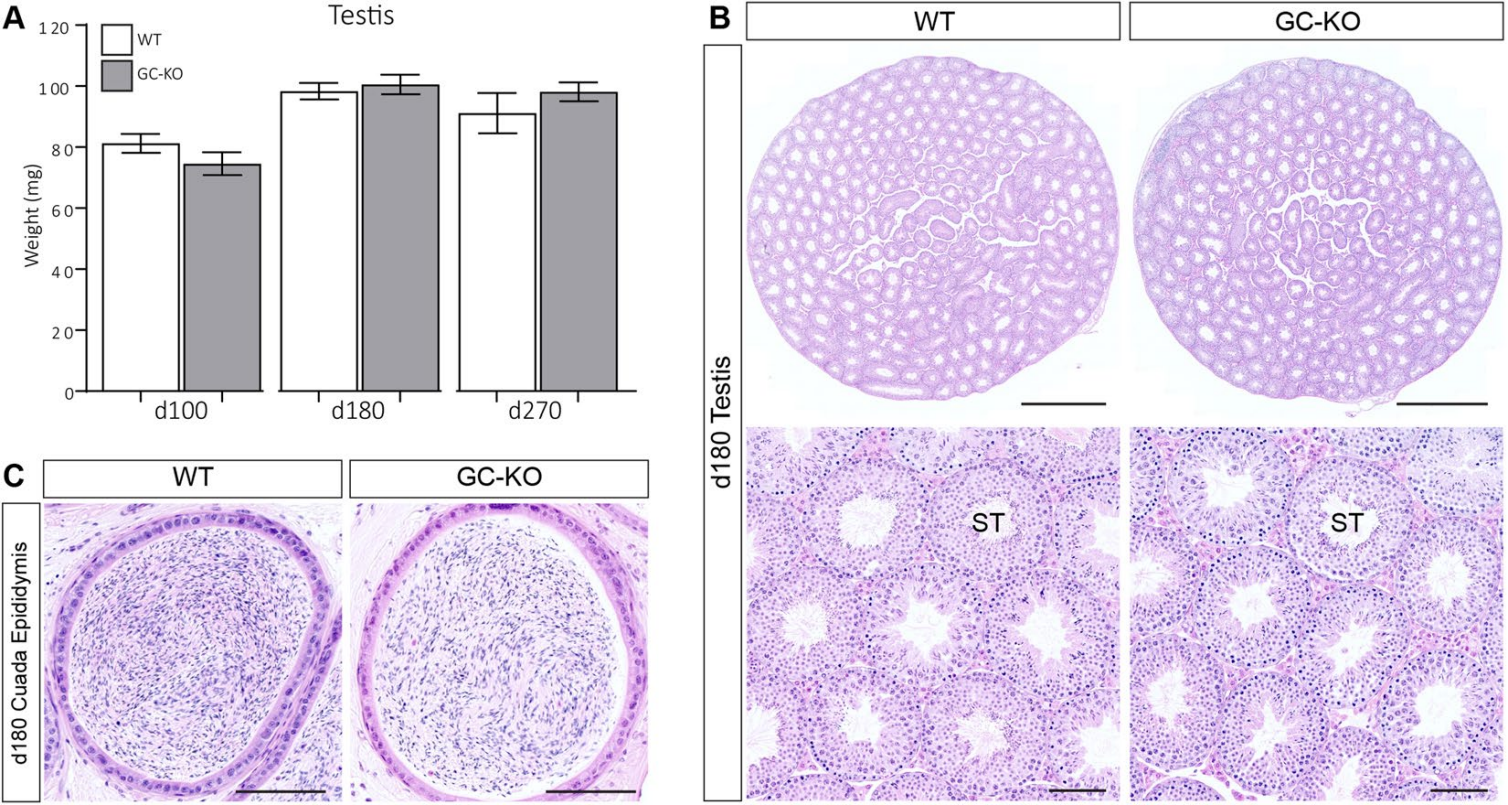
Testicular morphology is normal in GC-KO mice. **(A)** No difference in testis weight was observed between WT and GC-KO mice up to d270 (unpaired *t-*tests). Values are expressed as mean ± S.E.M of n = ≥10 mice per age/genotype. **(B)** Representative H&E staining of WT and GC-KO testes at d180. No difference in testis morphology was noted between WT and GC-KO mice. ST = seminiferous tubule, scale = 1 mm in top panels and 100 µm in bottom panels. **(C)** Representative H&E analysis of the cauda epididymis confirmed the presence of morphologically mature spermatids in both WT and GC-KO mice. Scale = 100 µm.

**Figure 5:**
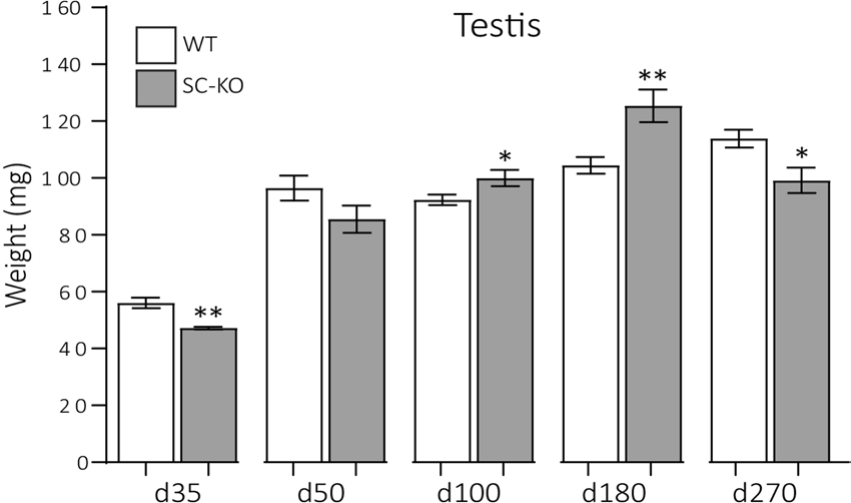
Testis weight is altered in SC-KO mice. At d35, a significant decrease in testis weight was observed in SC-KO animals (unpaired *t*-test; p = 0.0028, n = 7–9). At d50, testis weight was similar between WT and SC-KO animals (unpaired t-test; p = 0.1129, n = 9). At d100, a slight but significant increase in testis weight was observed in SC-KO mice (unpaired *t-*test; 0.0354, n = 13). This increase became more exaggerated at d180 (Mann-Whitney *U-*test; *p* = 0.0027; n = 12–16). By d270, a significant decrease in testis weight was observed in SC-KO mice (unpaired *t-*test; *p = *0.0195; n = 9–11). Values are expressed mean ± S.E.M.

**Figure 6:**
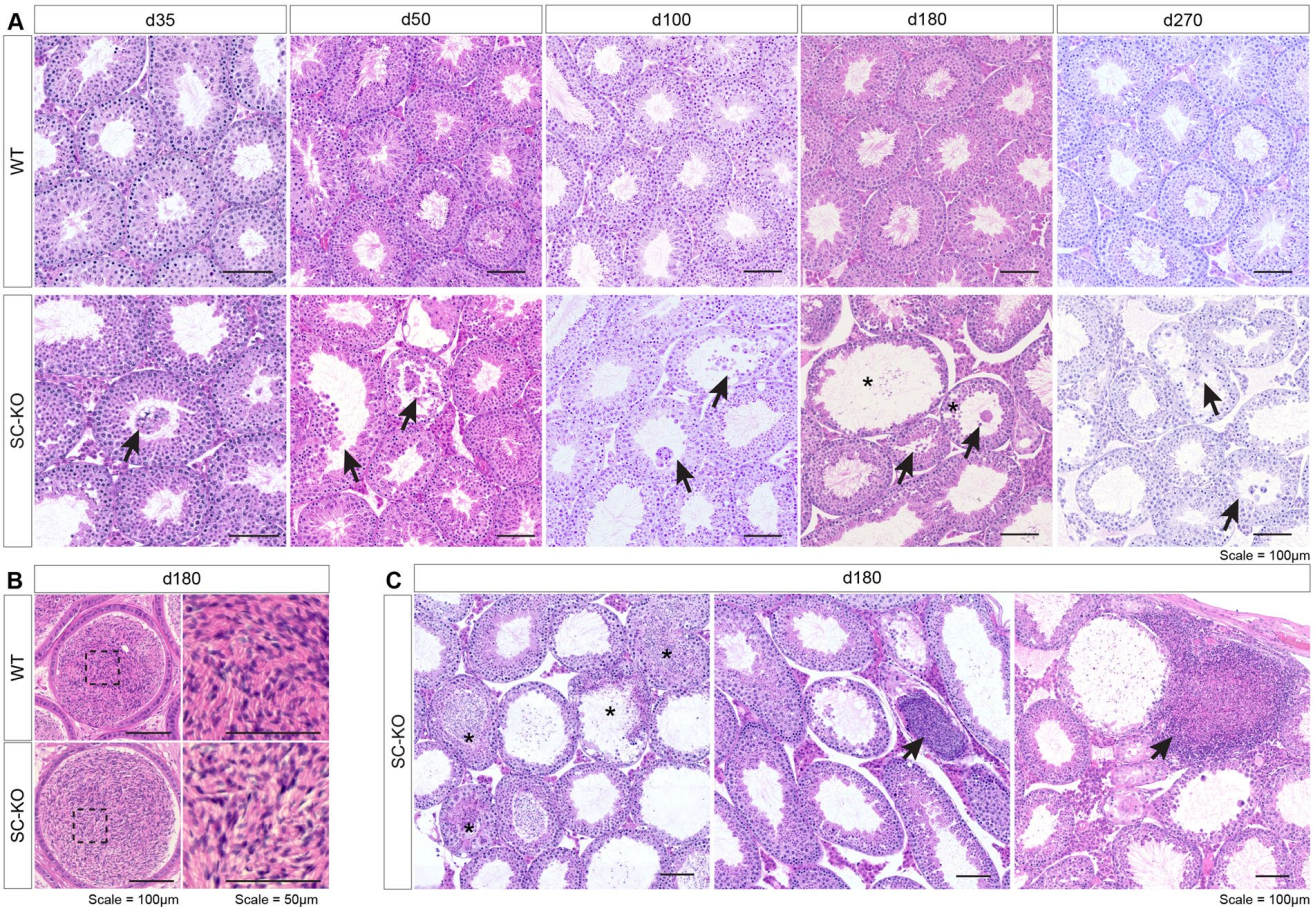
A Progressive degenerative testicular phenotype is observed in SC-KO mice. **(A)** Representative H&E staining of WT (top) and SC-KO testes (bottom) up to d270. Testis histology was largely normal in SC-KO up to d100 although evidence of germ cell sloughing was occasionally observed (arrows). At d180, more wide-spread degeneration of the seminiferous epithelium was observed in SC-KO testes. While morphologically normal tubules were present in the d180 SC-KO testis, a large proportion of tubules appeared distended and vacuolation of the seminiferous epithelium was noted (asterisks). **(B)** Morphologically mature spermatids were present in cauda epididymides of both WT and SC-KO mice at d180. **(C)** Serial sectioning through entire SC-KO testes revealed sections of tubule with increasing concentrations of sloughed germ cells/cellular debris (asterisks) and signs of apparent sperm stasis (arrows). Scale bars = 100 µm.

We next sought to characterise the testicular phenotype of our conditional *Lifr* mutants. We first assessed phenotypic changes in GC-KO animals. Testis weight did not differ between WT and GC-KO mice up to d180 (Fig. 4A) and no difference in testicular architecture was observed between WT and GC-KO mice (Fig. 4B). In addition, morphologically mature sperm were present in the cauda epididymides of both WT and GC-KO mice (Fig. 4C). These data unequivocally demonstrate that LIFR is not required in the germ cell population for qualitatively normal spermatogenesis to occur. Conversely, a striking testicular phenotype was observed in SC-KO animals. Initially, testis weight was reduced in SC-KO animals at d35 (Fig. 5), consistent with germ cell sloughing from the seminiferous epithelium, evidence of which was occasionally noted in SC-KO testes (Fig. 6A). Significant degeneration of the seminiferous epithelium was observed from d180 in SC-KO animals (Fig. 6A), accompanied first by increased testis weight at d180, followed by reduced testis weight at d270 (Fig. 5). While seminiferous tubule degeneration/atrophy was widespread, there were still tubules within SC-KO testes that appeared normal (Fig. 6A). Additionally, morphologically mature spermatids were present in both WT and SC-KO cauda epididymides at d180 (Fig. 6B), suggesting that LIFR-deficient Sertoli cells can still support all steps of spermatogenesis. From d180, the seminiferous tubules appeared dilated with larger lumens. When quantified, the luminal percentage volume was increased two-fold in the SC-KO testis and the percentage volume of the seminiferous epithelium was reduced by 25% compared to WT controls (Supplemental Fig. 1). Serial sectioning through entire SC-KO testes revealed regions of seminiferous tubule, often in close proximity to the rete testis, with increasing concentrations of sloughed germ cells/cellular debris and signs of apparent sperm stasis (Fig. 6C). The observed phenotype is consistent with increased fluid back-pressure inside the testis similar to that observed in several other rodent models^26–30^. To determine whether there is an additive effect of LIFR ablation from both germ cells and Sertoli cells together, we generated double SC-GC *Lifr* knockout mice. Testis histology in double mutants was similar to that of the SC-KO animals, further confirming that Sertoli cells, and not germ cells, are the major functional site of seminiferous tubule LIFR signalling (Supplemental Fig. 2).

### Sertoli cell and Spermatogonia Volume is Reduced in 6-month old SC-KO Mice

**Figure 7:**
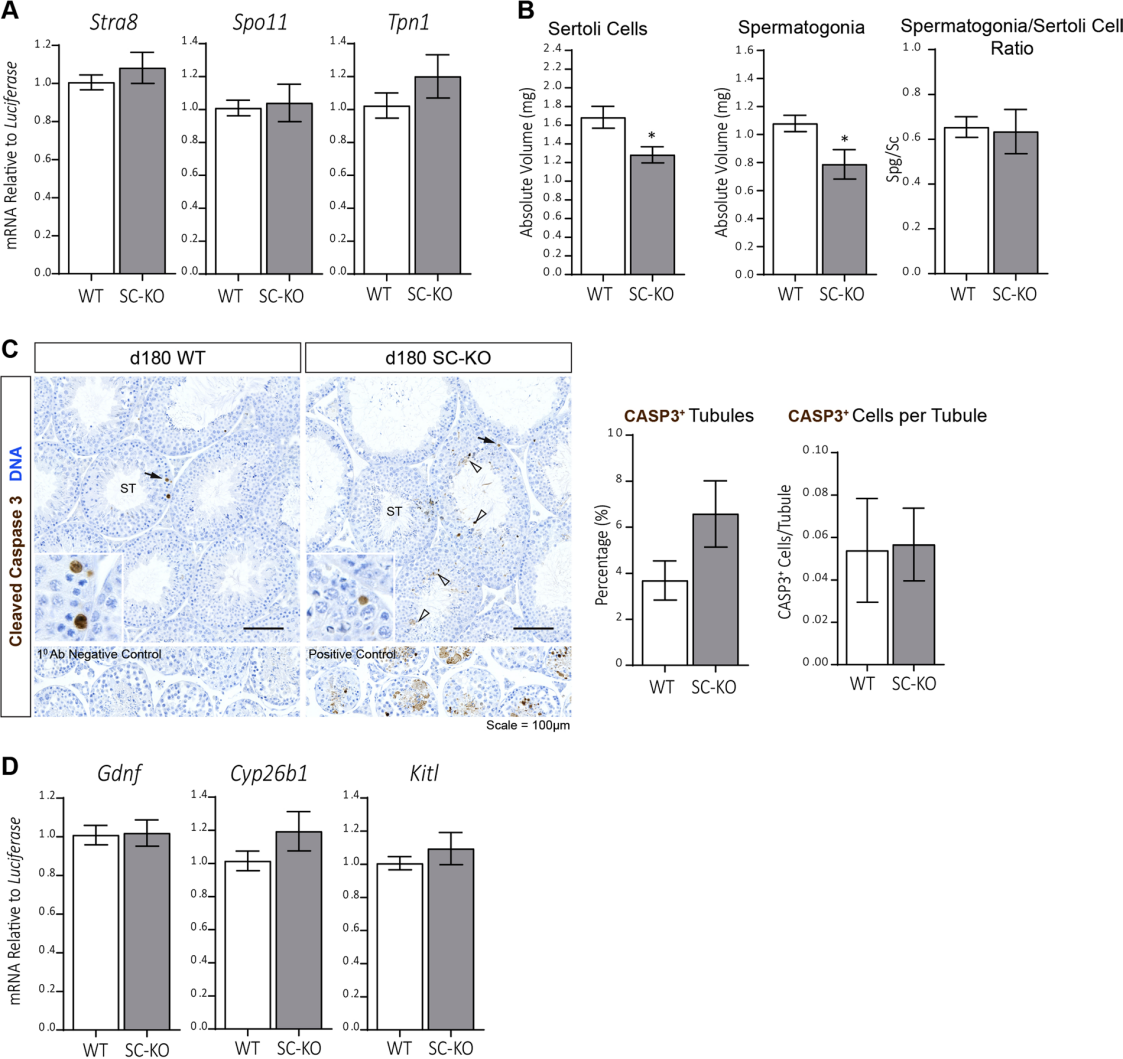
Reduced Sertoli cell and spermatogonia volume in SC-KO testes. **(A)** qRT-PCR analysis of spermatogonia (*Stra8*), spermatocyte (*Spo11*) and spermatid (*Tpn1*) specific mRNAs. Expression levels were similar between WT and SC-KO testes at d180 (unpaired *t-*test, n = 9–10). **(B)** Stereological analysis revealed a reduction in the absolute nuclear volume of Sertoli cells and spermatogonia in the SC-KO testis at d180 (unpaired *t-*test; *p* = 0.0172 and 0.0325 respectively; n = 7). Spermatogonia/Sertoli cell ratio is maintained in the SC-KO testis (unpaired *t-*test, n = 7). **(C)** Representative immunostaining for activated CASP3 (brown), as a marker of apoptosis, in WT and SC-KO testes at d180. No difference in the number of CASP3-positive tubules, or CASP3-positive cells lining the basal region of the tubules (black arrows, higher magnification insets) was observed between WT and SC-KO testes (unpaired *t-*test; n = 5–6). CASP3 immunoreactivity was occasionally observed throughout the seminiferous epithelium (open arrowheads) in SC-KO animals. Primary antibody negative control and experimentally-induced Sertoli cell death positive controls are included. Scale bars = 100 µm. **(D)** mRNA expression of *Gdnf, Cyp26b1* and *Kitl* was similar between WT and SC-KO testes (unpaired *t*-tests, n = 9–10). All values are expressed as the mean ± S.E.M.

To determine whether sloughing of a specific germ cell population was responsible for the tubule obstruction observed in SC-KO mice, we next measured the expression of germ cell-specific ‘biomarker’ mRNAs in WT and SC-KO testes. No difference in the expression of *Stra8*, *Spo11* or *Tpn1* was detected between WT and SC-KO testes at d180 (Fig. 7A), suggesting no major deficit in the spermatogonia, spermatocyte or spermatid populations respectively. However, stereological analysis of the seminiferous epithelium revealed a reduction in Sertoli cell and spermatogonia volume per testis in SC-KO mice at d180 by 24% and 27% respectively (Fig. 7B). The spermatogonia/Sertoli cell ratio was similar between SC-KO and WT controls (Fig. 7B), suggesting Sertoli cell support for spermatogonia is retained in SC-KO testes. No difference in spermatocyte or spermatid volume per testis was noted between WT control and SC-KO mice (Supplemental Fig. 3). We next asked whether increased apoptosis may explain the observed reduction in the number of Sertoli cells and spermatogonia. When quantified, no difference in the proportion of activated CASP3-positive tubules per section, or CASP3-positive cells in the basal region of seminiferous tubules was noted between WT and SC-KO animals (Fig. 7C). Finally, to determine whether a perturbation in the spermatogonial stem cell (SSC) niche may be responsible for the decreased number of spermatogonia, we measured the mRNA expression of Sertoli-cell derived factors which have been shown to be important for proper maintenance of SSCs^31^. Expression of *Gdnf, Cyp*2*6b1* and *Kitl* was similar in WT and SC-KO testes (Fig. 7D), suggesting that dysregulation of the SSC niche may not explain the reduction in spermatogonia in SC-KO testes.

### Blood-Testis-Barrier Function Remains Intact in the SC-KO Testis

**Figure 8:**
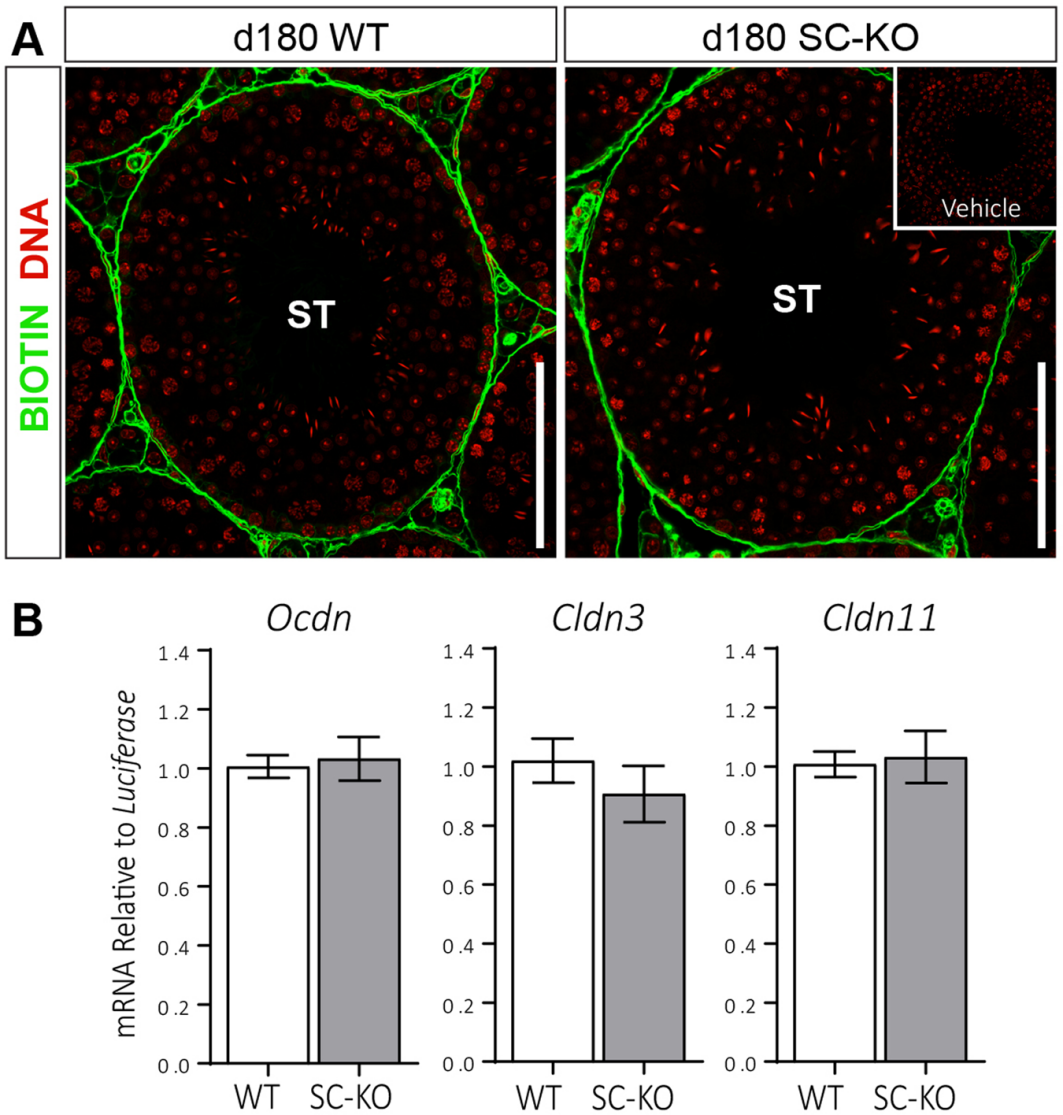
Blood-testis-barrier function remains functional in SC-KO mice. **(A)** A biotin tracer injected into the testis interstitium was restricted to the interstitial compartment between seminiferous tubules (ST) confirming the blood-testis-barrier remained intact in SC-KO mice at d180. Scale bars = 100 µm. **(B)** Expression of the blood-testis-barrier associated mRNAs *Ocdn*, *Cldn3* or *Cldn11* was unaltered in SC-KO testes at d180 (unpaired *t-*test; n = 9–10). Values are expressed as means ± S.E.M.

The blood-testis-barrier (BTB) plays an important role in maintaining the appropriate intratubular environment required for normal spermatogenesis. Previous reports have suggested a role for IL-6 family cytokines, of which LIF is a member, in the regulation of BTB function^32,33^. We therefore assessed the ability of LIFR-deficient Sertoli cells to maintain the integrity of the BTB. Functional assessment of the BTB using a biotin tracer confirmed the BTB remains intact in the SC-KO testis (Fig. 8A), consistent with normal mRNA expression of the BTB associated genes *Ocdn*, *Cldn3* or *Cldn11* (Fig. 8B).

## Discussion

Development and function of the mammalian testis is subject to endocrine regulation by the HPG axis, as well as a complex suite of paracrine factors, which ensure the adequate production of both sperm and androgens in adult males. In the present study, we set out to identify the contribution of leukemia inhibitory factor receptor (LIFR) signalling to testis development and function. Using a series of mutant *Lifr* mouse models, we found that LIFR does not appear to be crucial for prenatal testicular development. Conversely, LIFR in Sertoli cells, but not germ cells, is required for the maintenance of normal structure/function of the seminiferous tubules in adulthood.

Both *Lif* and *Lifr*, are expressed in the prenatal rodent testis from embryonic day (e) 12.5^18–20^ suggesting a role in testis organogenesis. In the present study, we observed a normal testicular architecture, characterised by fully formed seminiferous cords containing Sertoli and germ cells, and abundant interstitial fetal Leydig cells, in LIFR-deficient mice on the day of birth, suggesting that LIFR is dispensable for the establishment of a morphologically normal testis. Our analysis is limited to morphological assessment of testis structure at d0, thus we cannot rule out the possibility that LIFR-loss affects testicular organogenesis at earlier developmental time points, or in subtle ways which may not be readily apparent at d0. It has been reported previously that the number and morphology of primordial germ cells (PGC) is normal in LIFR-deficient mice when analysed at e10.5-13.5^23^, which would be consistent with our observations. Conversely, a slight reduction in PGC number was noted in gp130-deficient male fetuses^20^. Together, this suggests that further, more detailed analysis may be warranted. Whilst neither study made comment upon the developing somatic components of the fetal mouse testis, a recent study which aimed to identify genes involved in congenital abnormalities of the kidney and urogenital tract (CAKUT), noted a form of cryptorchidism in a CAKUT patient heterozygous for a hypomorphic *LIFR* mutation^34^. The authors suggest that disruption of murine *Lifr* also results in a potential form of cryptorchidism, characterised by an abnormal ligament attaching the testis to the dorsal aorta, present at e18.5. Such a defect was not noted in the present study, possibly owing to the genetic background of the *Lifr* mutants – in the above study animals were maintained on an outbred Ztm:NMRI background which may be more susceptible to developmental urogenital defects^35^. Alternatively, regression of this structure may occur between e18.5 and d0 – the time point at which our *Lifr* mutants were analysed. It may also be possible that such a defect went unnoticed during the course of the present studies. Nevertheless, our analysis of d0 testes demonstrate that LIFR is not required for the development of normal prenatal testicular structure.

Constitutive *Lifr* deletion results in a lethal phenotype prior to weaning thus LIFR deficient mice cannot be used for the study of adult testis development/function. We therefore used a Cre/*loxP* approach to disrupt *Lifr* in germ cells and Sertoli cells. Cre recombinase is reported to be active in spermatogonia from postnatal day 3 in the *TgStra8-icre*^*1Reb*^ mouse line^24^ which we used to disrupt *Lifr* in germ cells. In the rat testis, spermatogonia have been suggested to be a major target of LIFR signalling^18^, although this conclusion was arrived at through the comparison of d9 spermatogonia to d20 somatic cells. Surprisingly, analysis of our GC-KO mice suggests that LIFR is in fact dispensable in germ cells, from the spermatogonial stage onward, for normal spermatogenesis in mice. Functional redundancy between LIF and other IL6-family members signalling through gp130 provide a possible explanation, however, a previous report using *Tnap-cre* to delete gp130 from primordial germ cells reported no phenotype in mutant male mice^20^. However, no mention of the age of the animals was made in that study, thus the development of a progressive spermatogenic defect in ageing gp130-deficient animals cannot be ruled out.

A number of studies have documented wide-spread expression of *Lif* and *Lifr* in both germ and somatic cell populations in the adult rodent testis^18–21^, implicating LIF/LIFR signalling as a potential regulator of testicular function. Indeed, *in vitro* experiments have demonstrated that LIF can enhance the survival of gonocytes and Sertoli cells in a primary co-culture system^36^ and that LIF stimulates spermatogonial proliferation and/or survival when added to primary cultures of seminiferous tubule segments^18^. *In vitro* data suggest that LIF/LIFR signalling is active in rat Sertoli cells, but not germ cells^21^ indicating that the proliferative and/or anti-apoptotic effects of LIF on gonocytes/spermatogonia are likely mediated by the Sertoli cells present in the cultures. The data presented herein are consistent with this hypothesis, as we report that LIFR is dispensable in germ cells for normal spermatogenesis to occur, but Sertoli cell LIFR ablation appears to cause spermatogenic defects. Reduced numbers of spermatogonia in SC-KO testes was not be explained by increased apoptosis or obvious perturbation to the spermatogonial stem cell niche. Reduction of spermatogonia could be explained by altered proliferation of differentiating type A, intermediate and type B spermatogonia or may be secondary to reduced Sertoli cell numbers in the SC-KO testis. As we did not note any difference in apoptosis within the spermatogonia/Sertoli cell compartment of the seminiferous tubules of adult SC-KO mice, reduced Sertoli cell number could be due to altered proliferation during pubertal development, this requires further investigation.

Based on our histological analyses, postnatal testis development appears largely normal when LIFR is deleted from the Sertoli cell population. However, whilst LIFR-deficient Sertoli cells are able to support the production of mature spermatids, evidence of germ cell sloughing from the seminiferous epithelium was noted highlighting a requirement for LIFR for the maintenance of normal spermatogenesis. Despite an apparent reduction in Sertoli cells and spermatogonia, testis weight was significantly increased in d180 SC-KO mice and a large proportion of tubules appeared dilated. This is likely a result of increased fluid backpressure inside the testis due to occlusion of the testicular excurrent ductal system by sloughed germ cells^30,37^. However, the molecular mechanism(s) behind disruption to the seminiferous epithelium remains to be elucidated. Germ cell sloughing is often noted following toxicant-induced or genetic disruption to the Sertoli cell microtubule network^30,38–41^. Interestingly, the cytoplasmic form of signal transducer and activator of transcription 3 (STAT3; which is engaged following LIFR/gp130 signal transduction) has been implicated in the modulation of microtubule dynamics^42–44^. This raises the possibility that perturbed microtubule dynamics in LIFR-deficient Sertoli cells may in part explain premature germ cell exfoliation in SC-KO testes. Additionally, disruption of Sertoli cell microtubules results in decreased seminiferous tubule fluid production^45^ which could impair the efficiency of the excurrent ductal system resulting in blockage/sperm stasis as observed in SC-KO testes; this requires further investigation. Nevertheless, the studies conducted herein provide, to the best of our knowledge, the first *in vivo* evidence that LIFR signalling is required for normal testicular function in adulthood. Specifically, we have identified a requirement for Sertoli cell LIFR in the maintenance of normal spermatogenic function, further widening our understanding of the paracrine factors which support the function of this fundamentally important cell.

## Materials and Methods

### Ethics Statement

Experimental procedures and animal breeding and maintenance were approved by University of Edinburgh Animal Welfare and Ethical Review Body and were carried out with licenced permission under project licences 60/4200 and 70/8804 held by Professor Lee B. Smith in line with the UK Home Office Animals (Scientific Procedures) Act, 1986. Reference was made to the ARRIVE guidelines^46^ in the preparation of this manuscript.

### Transgenic Mice

Mice carrying a reporter-tagged *Lifr* knockout allele (*Lifr*^*tm1b(EUCOMM)Hmgu*^) were obtained from the IMPC project (http://www.mousephenotype.org/) and maintained on a C57Bl/6Ntac background. Constitutive *Lifr*-knockout mice were generated by intercrossing male and female *Lifr*^*wt/tm1b*^ mice to generate *Lifr*^*wt/wt*^, *Lifr*^*twt/tm1b*^ and *Lifr*^*tm1b/tm1b*^ offspring (herein referred to as WT, HET and KO respectively). Mice carrying a conditional *Lifr* allele (*Lifr*^*tm1c(EUCOMM)Hmgu*^) were also obtained from the IMPC project. Germ cell and Sertoli cell-specific *Lifr* knockout mice were generated by mating *Lifr*^*tm1c(EUCOMM)Hmgu*^ mice to mice expressing Cre-recombinase under the control of either the stimulated by retinoic acid-8 promoter (Stra8; *TgStra8-icre*^*1Reb*^)^24^ or Anti-Müllerian Hormone promoter (AMH; *TgAmh-cre*^*1Flor*^)^25^ respectively. First generation (F1) *Cre*^+/−^*; Lifr*^*wt/tm1c*^ and *Cre*^−/−^*; Lifr*^*wt/tm1c*^ were crossed to produce *Cre*^+/−^*; Lifr*^*tm1c/tm1c*^ and *Cre*^−/−^*; Lifr*^*tm1c/tm1c*^ animals in the second generation (F2; referred to as GC-KO and SC-KO respectively). Genomic DNA isolated from tail or ear biopsies was used to genotype mice by standard PCR techniques using either using either BioMix™ PCR reaction buffer (Bioline Reagents Ltd, UK) or Type-it Mutation Detect PCR Kit (QIAGEN Ltd., UK) according to the manufacturer’s instructions. Details of genotyping assays are listed in Table 1. *Lifr* recombination was assessed in genomic DNA isolated from testis biopsies of Cre^+^; *Lifr*^*wt/tm1c*^ mice. PCR products were analysed using the QIAxcel capillary electrophoresis system (Qiagen, UK).

**Table 1:** Details of genotyping assays.

**Assay**	**Forward Primer(s)**	**Reverse Primer(s)**	**Buffer**	**Product Sizes (bp)**
*Lifr* ^*tm1b*^	Lifr wt-fwd ggaaaccctggtattgtgga LacZ-fwd ccagttggtctggtgtca	Lifr wt-rev1 catgccacagtgcgacag	Type-it	*Lifr*^*wt*^ - 1150 *Lifr*^*tm1b*^ *-* 406
*Lifr* ^*tm1c*^	Lifr wt-fwd ggaaaccctggtattgtgga	Lifr wt-rev2 ggctgtcctggaactcactc	Type-it	*Lifr*^*wt*^ - 244 *Lifr*^*tm1c*^ - 418
*Lifr* ^*tm1d*^	5′ cas-fwd aaggcgcataacgataccac	3′ loxP-rev actgatggcgagctcagacc Lifr wt-rev2 ggctgtcctggaactcactc	Type-it	*Lifr*^*tm1c*^ - 389 *Lifr*^*tm1d*^ - 172
*Amh*-Cre	Amh-cre fwd cacatcaggcccagctctat Il2-fwd ctaggccacagaattgaaagatct	Amh-cre rev gtgtacaggatcggctctgc Il2-rev gtaggtggaaattctagcatcatcc	Biomix	*Amh-*cre - 180 *Il2* - 330
*Stra8*-Cre	Str8-cre fwd gtgcaagctgaacaacagga Il2-fwd ctaggccacagaattgaaagatct	Stra8-cre rev agggacacagcattggagtc Il2-rev gtaggtggaaattctagcatcatcc	Biomix	*Stra8-*cre - 260 *Il2* - 330

### Tissue Collection

Animals were sacrificed in the morning (8:00am - 11:00am) in accordance with Schedule 1 of the Animals (Scientific Procedures) Act, 1986. Exposure to an increasing concentration of carbon dioxide, followed by confirmation of permanent cessation of the circulation by palpitation was used for adult mice. Neonatal animals were sacrificed by destruction of the brain. Testis weights were recorded and tissues were either frozen on dry ice for storage at −80 °C or immersed in Bouin’s fixative (Clin-Tech Ltd, UK) for 6 hr. Bouin’s fixed tissues were processed, embedded in paraffin wax and sectioned at 5 µm for histological analyses.

### Histology and Immunostaining

For histological analyses, Bouin’s fixed, paraffin embedded tissue sections were stained with Haematoxylin and Eosin (H&E) following standard protocols. Testicular cell composition was estimated using standard stereological techniques involving point counting of cell nuclei to determine the absolute volume (mg) of Sertoli cell and germ cell nuclei^3^. Immuno-staining of an individual antigen of interest was performed using chromogenic immunostaining procedures as previously described^7^. For detection of multiple antigens on the same tissue section, multiplexed immuno-staining was performed using fluorogenic immunostaining procedures as described previously^47^. Details of the antibodies used are listed in Table 2. Images were acquired using either an LSM 780 confocal microscope (Carl Zeiss, UK) or an Axio Scan Z.1 slide scanner (Carl Zeiss, UK). CASP3-positive seminiferous tubules and CASP3-positive cells per tubule were scored from an average of 165 tubules across 2 separate testis sections per animal.

**Table 2:** Details of antibodies used for immunohistochemistry.

Target	Antigen Retrieval	Primary Antibody			Secondary Reagent			Detection Method
		Source	Catalogue No.	Dilution	Source	Cat. No.	Dilution	
HSD3b	Citrate pH 6	Santa Cruz Biotechnology *Germany*	sc30820	1:2000	Santa Cruz Biotechnology *Heidelberg, Germany*	sc-2961	1:200	ChαG HRP Tyramide
SOX9	Tris-EDTA pH 9	Merk Millipore *USA*	AB5535	1:4000	Vector Laboratories *Burlingame, CA, USA*	PI-1000	1:200	GαR HRP Tyramide
CASP3	n/a	Cell Signalling Technology *Netherlands*	9661 S	1:300	Anti-rabbit Poly-HRP-IgG *Leica Biosystems, UK*	DS 9800	n/a	Poly-HRP DAB
COUP-TFII	Citrate pH 6	Persus Proteomics *Japan*	PP-H7147-00	1:1000	Agilent Technologies (DAKO) *Cheadle, UK*	P0447	1:500	GαM HRP Tyramide
PDGFRb	Citrate pH 6	Abcam *UK*	ab32570	1:1500	Vector Laboratories *Burlingame, CA, USA*	PI-1000	1:200	GαR HRP Tyramide
DDX4	Citrate pH 6	Abcam *UK*	ab13840	1:1000	Vector Laboratories *Burlingame, CA, USA*	PI-1000	1:200	GαR HRP Tyramide

ChαG = Chicken anti-Goat; GαR = Goat anti-Rabbit; GαM = Goat anti-Mouse; HRP = Horseradish Peroxidase; DAB = 3, 3′-diaminobenzidine.

### Western Blotting

Western blotting for LIFR was performed as previously described^41^ with minor modifications. Briefly, proteins (30 µg) were separated on a 7% Tris-Acetate polyacrylamide gel (Thermo Fisher, UK) and transferred onto an Immobilon-Fl membrane (Merk Millipore, UK). Blots were probed with primary antibodies against LIFR (sc-659; 1:400; Santa Cruz Biotechnology, Germany) and TUBBA (ab6160; 1:5000; Abcam, UK). Primary antibodies were detected using Goat anti-Rabbit IRDye® 800CW (925-32211, 1:10,000; LI-COR Biosciences, UK) and Goat anti-Rat IRDye® 680RD (925-68076; 1:10,000; LI-COR Biosciences, UK) secondary antibodies. Blots were imaged using the LI-COR Odyssey imaging system (LI-COR biosciences, UK). Full blot images are included in supplementary materials.

### Quantitative RT-PCR

RNA was isolated from frozen testis tissue and cDNA synthesis was carried out as previously described^48^. Real-time PCR was carried using the ABI Prism 7900HT Real-Time PCR System (Applied Biosystems, UK), in 384-well format, with the Roche Universal ProbeLibrary (Roche, UK). qRT-PCR assays were designed using the Universal ProbeLibrary Design Centre tool (https://lifescience.roche.com/en_gb/brands/universal-probe-library.html). Details of the assays used are listed in Table 3. Data were analysed using the ΔΔCt method.

**Table 3:** Details of qRT-PCR assays.

**Gene**	**Forward Primer**	**Reverse Primer**	**UPL Probe**	**Efficiency (%)**
*Ocdn*	GCGGAAAGAGTTGACAGTCC	ATCTCCTGGGCCACTTCAG	25	99
*Cldn3*	TGGGAGCTGGGTTGTACG	CAGGAGCAACACAGCAAGG	26	99
*Cldn11*	TGGAGTGGCCAAGTACAGG	GACAATGGCGCAGAGAGC	20	93
*Stra8*	TTGACGTGGCAAGTTTCCT	AGTTGCAGGTGGCAAACATA	107	102
*Spo11*	GGCTCCTGGACGACAACTT	CAGATCTGGAACGCCCTTT	18	99
*Tpn1*	AGCCGCAAGCTAAAGACTCA	CGGTAATTGCGACTTGCAT	91	96
*Gdnf*	GCTCAAAATTGTGACAACCTCA	CAGAGGGTCTGGAACGACAT	107	104
*Cyp26b1*	AACATGGCAAGGAGATGACC	TTGCATGATCAAGGATGTGC	17	99
*Kitl*	GCTGCTGGTGCAATATGCT	GATAAATGCAAGTGATAATCCAAGTTT	50	97

### Functional Integrity of the Blood-Testis-Barrier

Permeability of the blood-testis-barrier to a biotin tracer was carried out as previously described^49^ with minor modifications. Briefly, EZ-link sulfo-NHS-LC-biotin (Thermo Scientific, UK) was freshly prepared as a 10 mg/mL solution in 0.01 M MgCl2 in PBS. 10–15 uL of biotin solution was injected into the testis under the tunica albuginea immediately after sacrifice, 10–15 uL of 0.01 M MgCl2 in PBS was injected into the contralateral testis as a control. Testes were left on ice for 30 mins and then fixed and processed as described above. The localisation of the biotin tracer was visualised by incubation with Streptavidin Alexa Fluor™ 546 conjugate (S11225, Thermo Scientific, UK), diluted 1:200 in PBS, for 1 hr at room temperature. Tissue sections were imaged using an LSM 780 confocal microscope (Carl Zeiss, UK).

### Statistical Analyses

Data were statistically analysed using GraphPad Prism 7.02 software (GraphPad software Inc., USA). The D’Agostino & Pearson or Shapiro-Wilk normality test was first used to assess the distribution of data. Comparisons were made between two groups using either an unpaired, two tailed, *t*-test (for parametric data) or the Mann-Whitney *U*-test (for non-parametric data). Differences between more than two groups conforming to a Gaussian distribution were identified using a one-way analysis of variance (ANOVA). For one-way ANOVA, means were compared between groups using Dunnet’s post hoc analysis. Transgene inheritance was assessed using a Chi-square test. In each case, a *p*-value ≤ 0.05 was considered statistically significant.

## Electronic supplementary material

Supplementary Information
