# Supplementary material for: Leukemia Inhibitory Factor-Receptor is Dispensable for Prenatal Testis Development but is Required in Sertoli cells for Normal Spermatogenesis in Mice
Source: Sci Rep. 2018 Aug 1;8:11532. doi: 10.1038/s41598-018-30011-w (PMC6070476; doi:10.1038/s41598-018-30011-w)
Supplement: Supplementary file 1 — Supplementary Information [file 41598_2018_30011_MOESM1_ESM.pdf]

Leukemia Inhibitory Factor-Receptor is Dispensable for Prenatal Testis Development but is Required in Sertoli cells for Normal Spermatogenesis in Mice.

Michael Curley[1], Laura Milne[1], Sarah Smith[1], Nina Atanassova[2], Diane Rebourcet[1], Annalucia Darbey[1], Patrick W.F. Hadoke[3], Sara Wells[4] and Lee B. Smith[1, 5*]

[1] MRC Centre for Reproductive Health, University of Edinburgh, The Queen’s Medical Research Institute, 47 Little France Crescent, Edinburgh, EH16 4TJ, United Kingdom.

[2] Institute of Experimental Morphology, Pathology and Anthropology with Museum, Bulgarian Academy of Sciences, 1113, Sofia, Bulgaria

[3] The British Heart Foundation Centre for Cardiovascular Science, University of Edinburgh, The Queen's Medical Research Institute, Edinburgh, EH16 4TJ, United Kingdom.

[4] Mary Lyons Centre, MRC Harwell, Harwell Campus, Oxfordshire, OX11 ORD, United Kingdom.

[5] School of Environmental and Life Sciences, University of Newcastle, Callaghan, NSW 2308, Australia.

[*] Correspondence and requests for materials: Professor Lee Smith, MRC Centre for Reproductive Health, University of Edinburgh, The Queen’s Medical Research Institute, 47 Little France Crescent, Edinburgh, EH16 4TJ, United Kingdom.

e-mail: lee.smith@ed.ac.uk

Supplementary Information

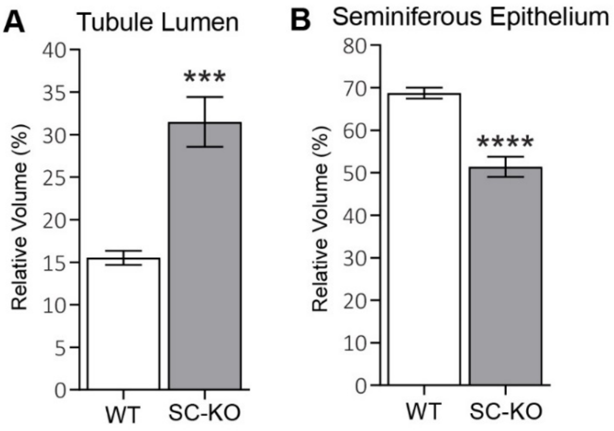

Supplemental Figure 1. Altered seminiferous tubule lumen and seminiferous epithelium in SC-KO testes. A two-fold increase in percentage volume of the tubule lumen **(A)** and a 25% reduction in the seminiferous epithelium **(B)** was observed when *Lifr* is disrupted in Sertoli cells (Unpaired *t*-tests; *** p = 0.0002 and **** p = <0.0001 respectively, n = 7).

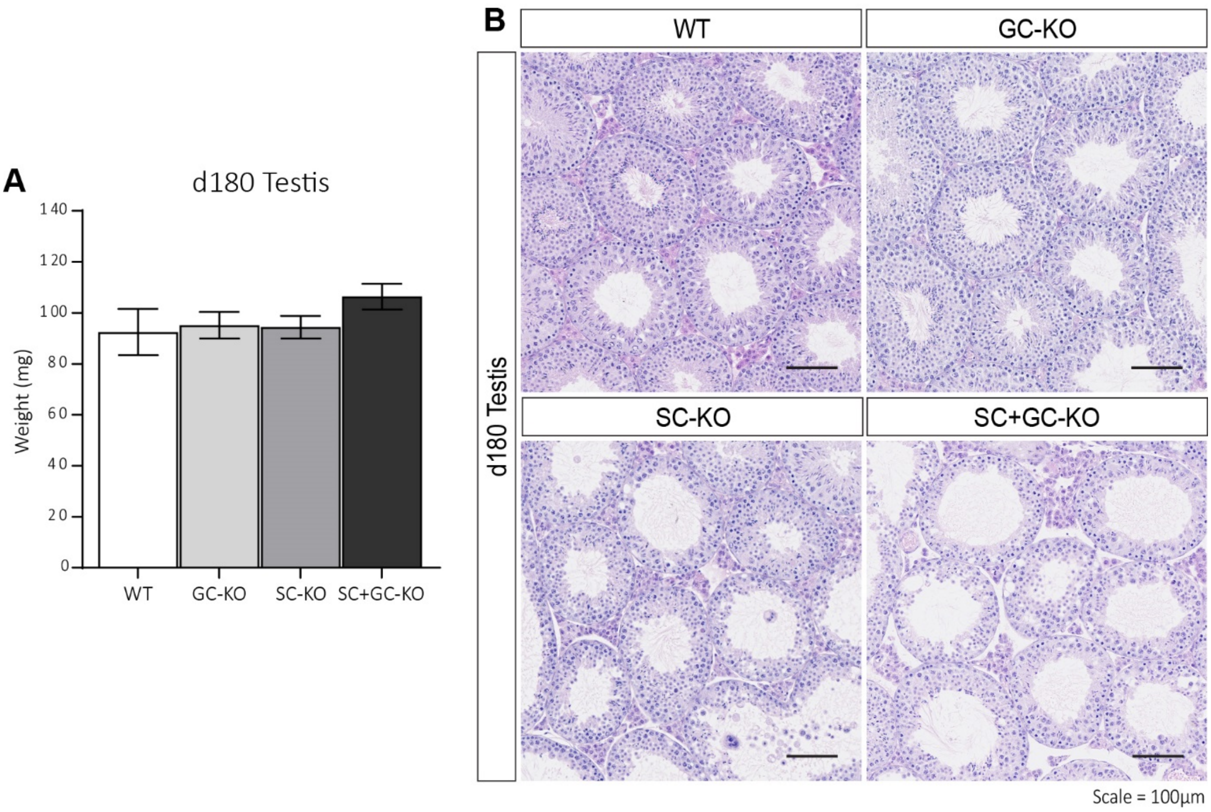

Supplemental Figure 2. No additional effect is observed when *Lifr* is disrupted in both germ cells and Sertoli cells. (A) No significant difference in testis weight at d180 was observed when *Lifr* is disrupted in both germ cells and Sertoli cells (GC+SC-KO; 1-way AVOVA, n = 6-13). **(B)** Representative H&E staining of WT, GC-KO, SC-KO and GC+SC-KO testes at d180. Testicular phenotype was similar in both SC-KO and GC+SC-KO confirming that *Lifr* is dispensable in germ cells but not Sertoli cells for normal spermatogenic function. Scale = 100μm.

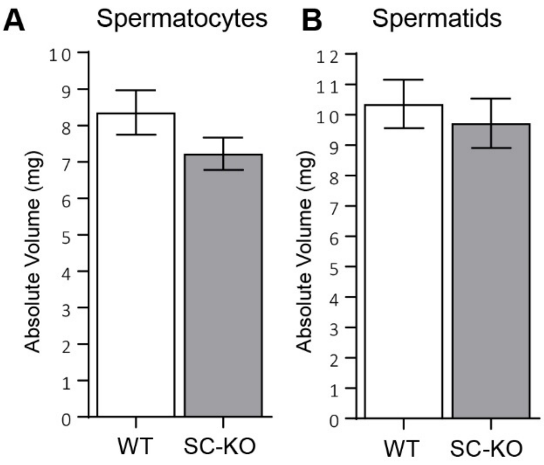

Supplemental Figure 3. Spermatocyte and spermatid volume is unaltered in SC-KO testes. No difference in **(A)** spermatocyte or **(B)** spermatid volume per testis was observed between WT and SC-KO mice (unpaired *t*-test; n = 7).

Full images of blots from figures 1C and 3F and G.

Western Blot from Fig. 1

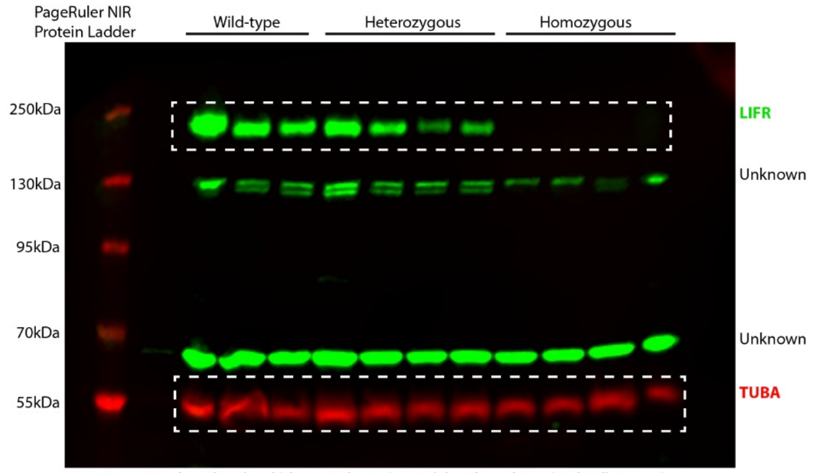

7% Tris-Acetate polyacrylamide gel (Thermo Fisher, UK)/Immobilon-Fl membrane (Merk Millipore, UK)
Whole Neonatal Brain Protein Exrtact

Western Blots from Fig. 3

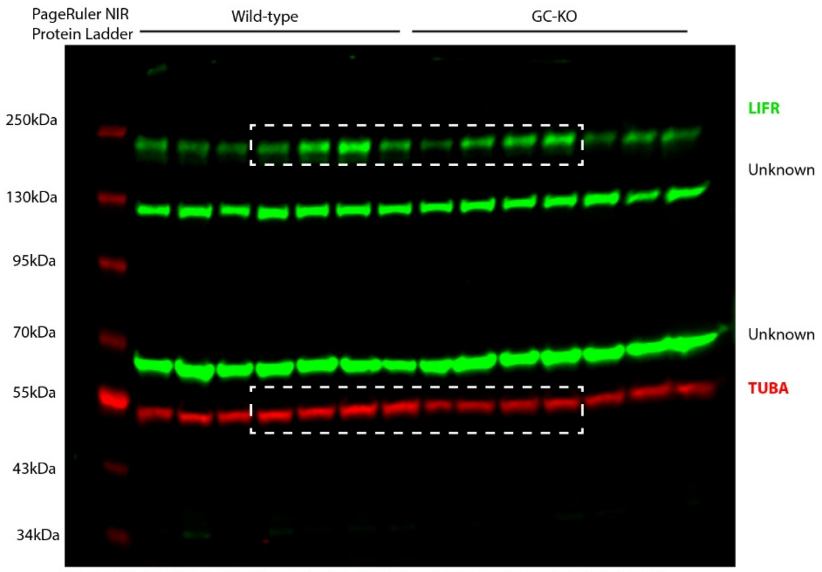

7% Tris-Acetate polyacrylamide gel (Thermo Fisher, UK)/Immobilon-Fl membrane (Merk Millipore, UK)
Adult Testis Protein Exrtact

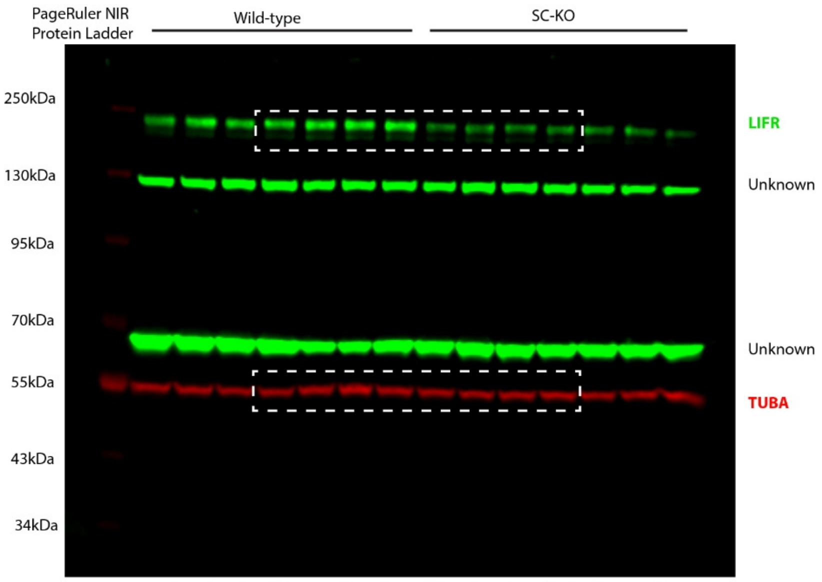

7% Tris-Acetate polyacrylamide gel (Thermo Fisher, UK)/Immobilon-Fl membrane (Merk Millipore, UK)
Adult Testis Protein Exrtact
